# Identification of Novel Phenotypes Correlated with CKD: A Phenotype-Wide Association Study

**DOI:** 10.7150/ijms.63973

**Published:** 2022-10-31

**Authors:** Yifen Lin, Jianqiu Kong, Ting Tian, Xiangbin Zhong, Shaozhao Zhang, Haojin Zhou, Zhenyu Xiong, Jiashu Zhao, Yiquan Huang, Menghui Liu, Yuehua Dong, Junjiong Zheng, Xiayao Diao, Jieyin Wu, Haide Qin, Yue Hu, Xueqin Wang, Xiaodong Zhuang, Xinxue Liao

**Affiliations:** 1 Cardiology department, First affiliated hospital of Sun Yat-Sen University.; 2 NHC Key Laboratory of Assisted Circulation (Sun Yat-Sen University).; 3 Department of Urology, Sun Yat-Sen Memorial Hospital, Sun Yat-Sen University.; 4 Guangdong Provincial Key Laboratory of Malignant Tumor Epigenetics and Gene Regulation, Sun Yat-Sen Memorial Hospital.; 5 Department of Statistical Science, School of Mathematics, Southern China Center for Statistical Science, Sun Yat-Sen University.; 6 Department of Urology, the Third Affiliated Hospital of Sun Yat-Sen University.

**Keywords:** chronic kidney disease, phenotype-wide association study, retinol, red cell distribution width, C-peptide.

## Abstract

**Background:** A comprehensive understanding of phenotypes related to CKD will facilitate the identification and management of CKD. We aimed to panoramically test and validate associations between multiple phenotypes and CKD using a phenotype-wide association study (PheWAS).

**Methods:** 15,815 subjects from cross-sectional cohorts of the National Health and Nutrition Examination Survey (1999-2006) were randomly 50:50 split into training and testing sets. CKD was defined as eGFR < 60 mL/min/1.73m^2^. We performed logistic regression analyses between each of 985 phenotypes with CKD in the training set (false discovery rate < 1%) and validated in the testing set (false discovery rate < 1% ). Random forest (RF) model, Nagelkerke's Pseudo-R^2^, and the area under the receiver operating characteristic (AUROC) were used to validate the identified phenotypes.

**Results:** We identified 18 phenotypes significantly related to CKD, among which retinol, red cell distribution width (RDW), and C-peptide were less researched. The top 5 identified phenotypes were blood urea nitrogen (BUN), homocysteine (HCY), retinol, parathyroid hormone (PTH), and osmolality in RF importance ranking. Besides, BUN, HCY, PTH, retinol, and uric acid were the most important phenotypes based on Pseudo-R^2^. AUROC of the RF model was 0.951 (full model) and 0.914 (top 5 phenotypes).

**Conclusion:** Our study demonstrated associations between multiple phenotypes with CKD from a holistic view, including 3 novel phenotypes: retinol, RDW, and C-peptide. Our findings provided valid evidence for the identification of novel biomarkers for CKD.

## Introduction

Chronic kidney disease (CKD) is a worldwide public health problem which results in adverse clinical outcomes, such as cardiovascular disease, kidney failure and death 1. Currently, CKD affects 8% to 16% of the world's population and accounts for 16.05 deaths per 100,000 people 2, 3. A comprehensive understanding of phenotypes related to CKD will facilitate the control of CKD, both in disease identification and management. However, the research on the association between risk factors and CKD often focused on single factors 4-6, which may be subject to selection biased and false positive reporting and finally lead to an incomplete understanding of possible risk factors and pathogenesis of CKD.

To address these concerns, we applied the “phenotype-wide association study (PheWAS)”, a high-throughput methodology analogous to the genome-wide association study (GWAS), to comprehensively search for new risk factors associated with disease 7-10. From a holistic and unbiased perspective like GWAS, the PheWAS can evaluate multiple risk factors instead of only one factor at one time, which helps to overcome the selective reporting bias and false positive reporting 11, 12. In recent years, a similar systematic approach has been used to explore the association between numerous exposomes and outcomes 7, 10, 13, 14.

The objective of this investigation is to systematically search 985 phenotypes with respect to CKD using the National Health and Nutrition Examination Survey (NHANES) from years 1999-2006. We also investigate the prioritization and diagnostic accuracy of the identified phenotypes of CKD. We believe that using PheWAS to assess environment and lifestyle factors in CKD will provide a richer understanding of the architecture of complex traits.

## Method

### Study population

We use data from the National Health and Nutrition Examination Survey (NHANES), a public access database constructed by the US Centers for Disease Control and Prevention (CDC). The NHANES recruited US civilian, non-institutionalized civilian residents every two years and collected participants' information via in-person interviews, physical examinations, and laboratory data 15. In present study, we included 15,815 individuals aged > 20 years from four cohort survey (1999-2000, 2001-2002, 2003-2004, 2005-2006). The National Center for Health Statistics ethics review board approved the conduct of NHANES, and participants gave written informed consent. All methods were carried out in accordance with the approved guidelines. The analysis was deemed exempt by the CDC Institutional Review Board.

### Outcome definition

Chronic kidney disease (CKD) is defined as glomerular filtration rate (GFR) < 60mL/min/1.73m^2^. And GFR can be measured as the renal clearance of exogenous filtration markers indirectly. The eGFR was calculated using the Chronic Kidney Disease Epidemiology Collaboration (CKD-EPI) formula [for women with Scr ≤0.7, (Scr/0.7)^-0.329^ × (0.993)^age^ (× 166 if black, × 144 if white or other); for women with Scr >0.7, (Scr/0.7)^-1.209^ × (0.993)^age^ (× 166 if black, × 144 if white or other); for men with Scr ≤0.9; (Scr/0.9)^-0.411^ × (0.993)^age^ (× 163 if black, × 141 if white or other); for men with Scr >0.9, (Scr/0.9)^-1.209^ × (0.993)^age^ (× 163 if black, × 141 if white or other)] in subjects > 40 years of age 16. We used the NHANES-recommended calibrations for serum creatinine measurements.

### Phenotypes in the PheWAS

A total of 1,181 phenotypes were collected in the original dataset, and those with sample size less than 500 and most observations (> 90%) less than detection threshold were excluded, remaining 985 phenotypes in the final analysis (Table S1). We categorized the phenotypes into 19 classes according to NHANES categorization: 31 body measurements, 19 of blood routine, 76 of biochemistry, 42 of nutritional status, 78 of urine test, 38 of infection status, 39 on disease history, 204 on drugs used, 46 on lifestyle, 14 on living condition, 208 on dietary, 14 on drug addiction, 29 of dioxins, 35 of polychlorinated biphenyls (PCBs), 34 of pesticides, 9 of phenols, 15 of polybrominated diphenyl ethers (PBDE), 11 of polyfluorinated compounds and 43 of volatile organic compounds. The measuring methods of all the variables were published at https://wwwn.cdc.gov/nchs/nhanes/default.aspx.

### Statistical analysis

**Figure 1:**
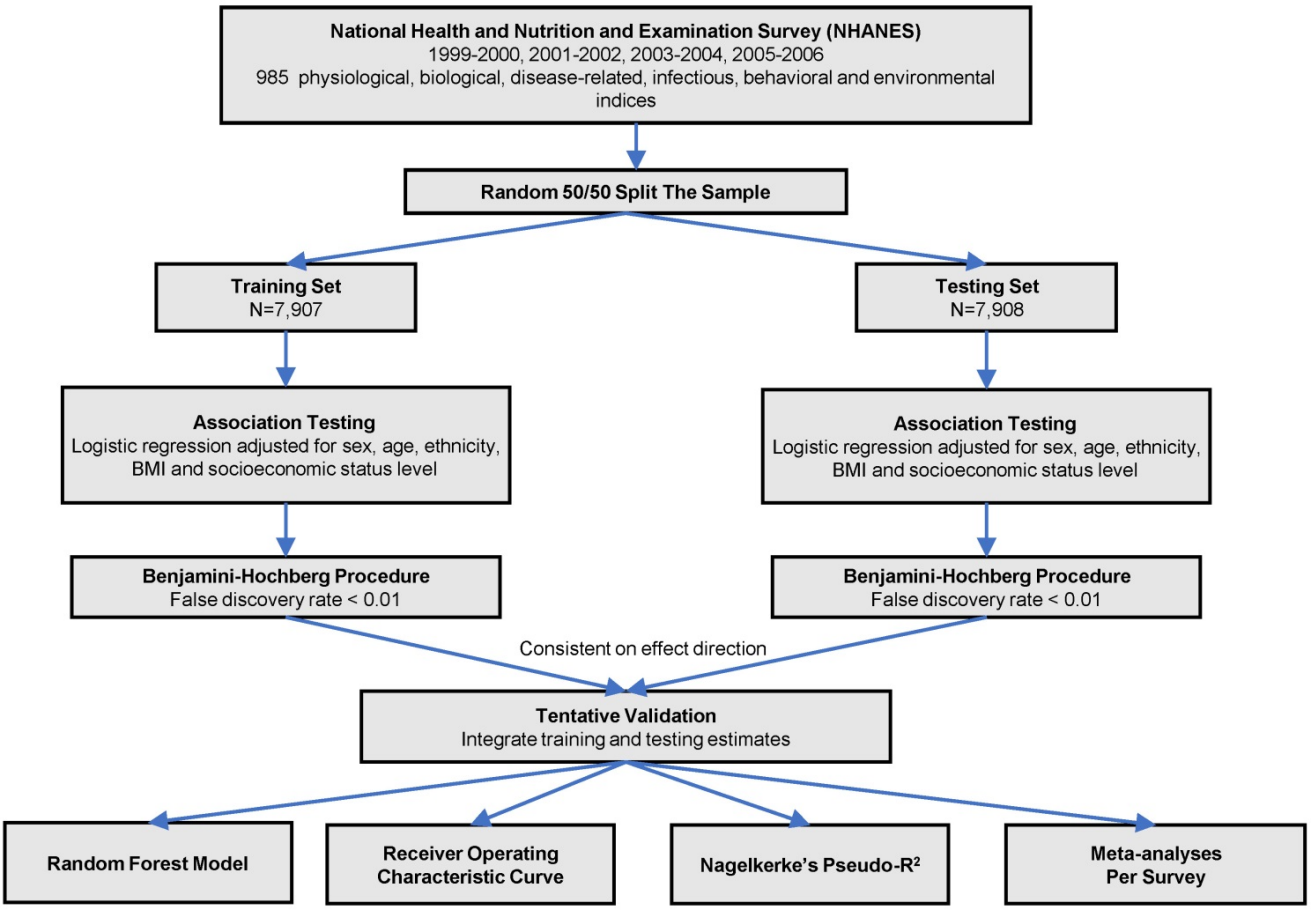
Study flow chart of phenotype-wide association study statistical methods.

The analytic procedure is depicted in Figure 1. We performed the Kolmogorov-Smirnov test to evaluate the distributions of continuous variables. Those with *P* < 0.05 were considered as skewed distribution and log transformed. All the continuous variables were z-standardized. To valid our result within the dataset, we did a random 50:50 split of the dataset into training set and testing set. Baseline characteristics in training and testing sets were demonstrated using one-way ANOVA or the Kruskal-Wallis test forcontinuous variables and 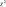 tests for categorical variables. We used logistic regression models to analyze the associations between phenotypes with CKD adjusted for sex, age, ethnicity, body mass index and socioeconomic status (SES) level in two sets. We estimated the false discovery rate (FDR) via the Benjamin-Hochberg procedure to control for the proportion of significant results that are false positives due to errors. We supposed a phenotype significant as follows: (1) FDR < 1% in the training set; (2) FDR <1% in the testing set, only those phenotypes moved forward from the training set were taken into consideration; (3) Consistent direction of effect in both training and testing set. We computed the odds ratio (OR) and 95% confidence interval (CI) for the tentatively validated phenotypes.

All the subsequent analyses were performed in the combined dataset of training and testing sets. We evaluated the Pearson correlations among all identified phenotypes and presented their correlation in a heat map. The more considerable correlation between a pair of variables, the deeper color shown in the graph. We then analyzed the identified phenotypes using a random forest (RF) model for multicollinearity elimination and ranked the phenotypes based on variable importance scores (increase in node purity). RF model was performed with 1000 decision trees, using the “randomForest” R package. Also, we assess the discriminative power of the identified phenotypes for CKD by receiver operating characteristic (ROC) curves. Additionally, we calculated Nagelkerke's Pseudo-R^2^ to evaluate the variance of the outcome explained by validated phenotypes. Moreover, a random-effect META-analysis, graphically represented using forest plots, was performed to combine the 1999-2000, 2001-2002, 2003-2004, 2005-2006 surveys to increase power for discovery and evaluate the heterogeneity between survey year. Finally, for three novel phenotypes identified, we performed subgroup analyses using logistic regression model stratified by gender, race, BMI, age, hypertension and diabetes.

All statistical tests were performed in R version 3.5.1 (The R Foundation for Statistical Computing, www.R-project.org).

## Results

### Baseline characteristics

Baseline characteristics of 15,815 participants with or without CKD in the training and testing dataset are shown in Table 1. A total of 1,355 (8.6%) individuals were diagnosed as CKD, with 678 (8.6%) and 677 (8.6%) individuals in the training and testing sets, respectively. Individuals with CKD tended to be old, white, and have lower SES level in both groups. Besides, individuals with history of diabetes or hypertension were more likely to have CKD.

### Association between phenotypes with CKD

**Figure 2:**
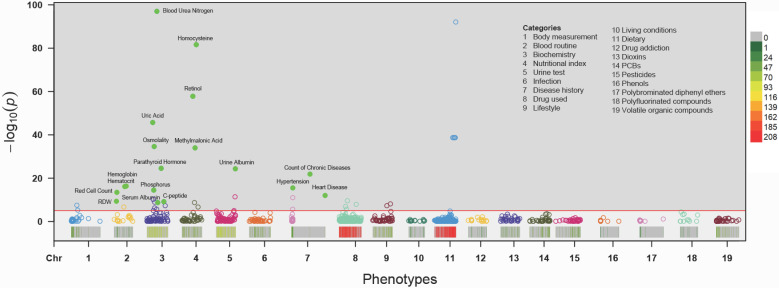
Manhattan plot showing the phenotype-wide association with chronic kidney disease in the NHANES cohorts. Y-axis presents -log10 (P value) of the adjusted logistic regression model for each of the phenotypes. Horizontal line indicates the level of significance corresponding to the false discovery rate less than 1%. Each x-axis label indicates a variable category and within each category, the interval to the label represents standardized odds ratio (OR) for each phenotype. Filled marks represent tentatively validated phenotypes in the testing set (p < 0.01). Analyses are adjusted for age, sex, ethnicity, body measure index and socioeconomic status (SES) level.

Associations for the 985 phenotypes with CKD adjusted for age, sex, BMI, ethnicity and socioeconomic position in the total sample are shown in a Manhattan plot with *P* values on the -log 10 scale (Figure 2). Eighteen variables showed an FDR <1% in the training set and were tentatively validated (FDR <0.01) in the testing set. The odds ratios (ORs) and 95% confident intervals (95%CIs) of identified phenotypes were shown in Table 2. In the training set, a higher level of red cell distribution width (RDW), blood urea nitrogen (BUN), uric acid (UA), osmolality, parathyroid hormone (PTH), C-peptide, homocysteine (HCY), retinol, methylmalonic acid (MMA) and urine albumin were associated with increased risk of CKD. High prevalence of hypertension, diabetes mellitus, and other chronic disease (detailed definition in Text S1) also showed a positive correlation with CKD. Meanwhile, inverse associations between hemoglobin, hematocrit, red cell count, and serum albumin with CKD were also substantiated in the training set. Besides, the effects of identified phenotypes in training set were similar to testing set. Besides, the associations between retinol, RDW, and C-peptide with CKD in subgroup analyses were consistent (Table S2-4).

### Correlation patterns of identified phenotypes

As shown in Figure S1, the correlations among the validated phenotypes were evaluated using Pearson correlations and presented in a heatmap. Most phenotypes showed weak or zero correlations with each other (Pearson Correlation Coefficient ρ < 0.4). Modest to strong correlations were observed among some phenotypes which belong to the same categories or share similar clinical implications. It is worth noting that hemoglobin, hematocrit, and red cell count were strongly correlated with each other, with correlation coefficient around 0.8.

### Ranking of identified phenotypes

**Figure 3:**
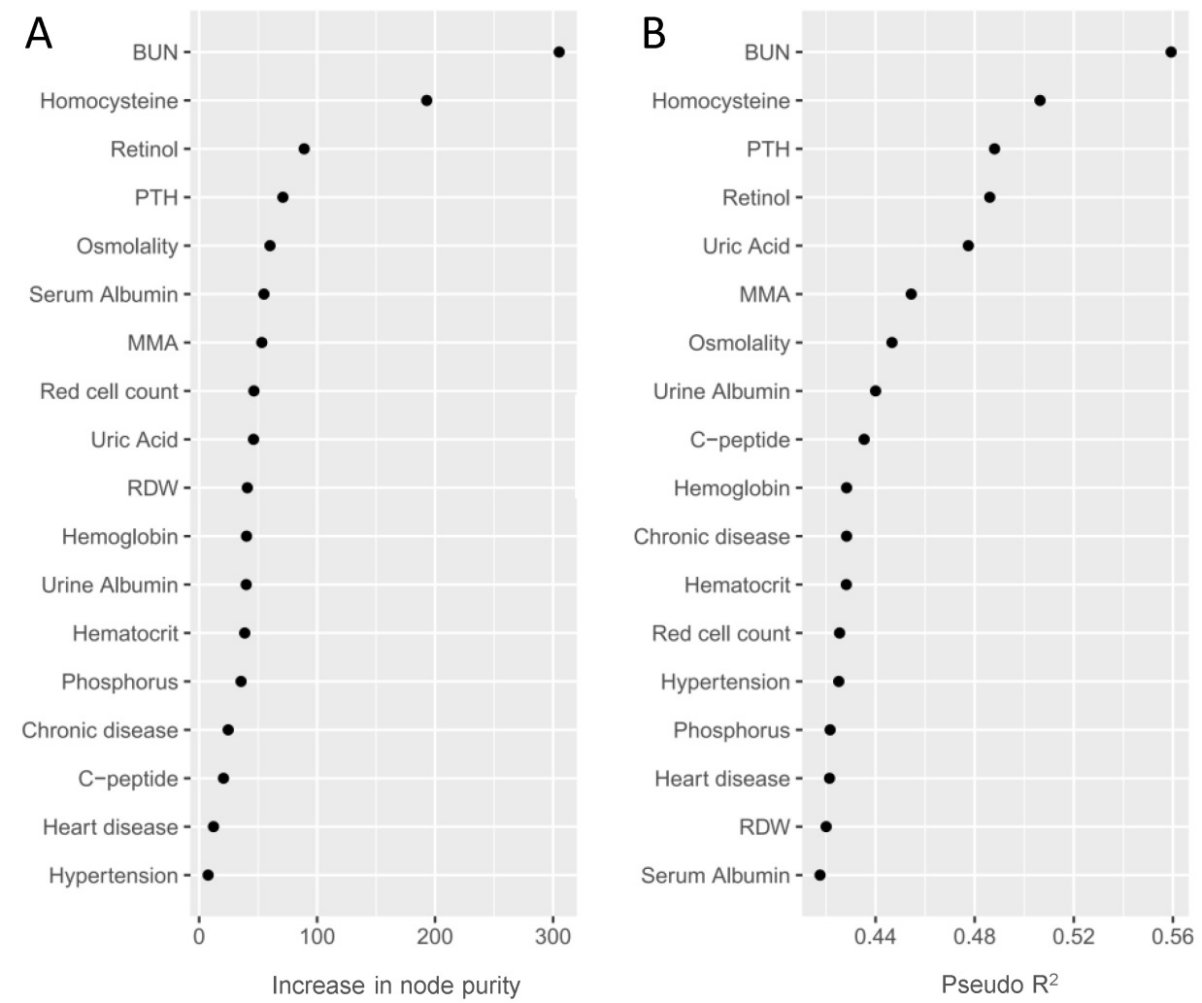
Random forest importance ranking (A) and Nagelkerke's Pseudo-R^2^ ranking (B) of the validated phenotypes. Random forest importance ranking (via increase in node purity) indicates the discriminative performance of the identified phenotypes for CKD. Nagelkerke's Pseudo-R^2^ demonstrates the variance of the outcome explained by validated phenotypes.

We used a RF model to eliminate multicollinearity and rank the identified phenotypes according to their classification performance. As shown in Table 2 and Figure 3, we prioritized the identified phenotypes based on variable importance scores: BUN, HCY, retinol, PTH, osmolality, serum albumin, MMA and so on. Besides, we also rank the identified phenotypes using Nagelkerke's Pseudo-R^2^. We observed that BUN and HCY were the most influential phenotypes to CKD, followed by PTH, retinol, uric acid, MMA, osmolality and so on (Table 2 and Figure 3).

### Diagnostic efficacy of phenotypes on CKD

We used the areas under the ROC curve (AUROC) to evaluate the discriminative power of phenotypes identified (Table 2 and Figure S2). We omitted hemoglobin and hematocrit in the incorporated model for their strong correlation and collinearity with red cell count. The AUROC ranged from 0.529 (phosphorus) to 0.877 (BUN and homocysteine) for single phenotype. The AUROC for the model incorporated all phenotypes, and top 5 phenotypes in the RF model except BUN were 0.951 and 0.914, respectively (Figure S2).

### META analysis of identified phenotypes

To further validate the effects of identified phenotypes, we assessed the heterogeneities using a random-effect meta-analysis in 1999-2000, 2001-2002, 2003-2004, 2005-2006 surveys based on survey-weighted logistic regression models (Figure S3). We found that most of the phenotypes have mild to moderate heterogeneity (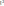
< 50%) in different surveys. However, we observed obvious heterogeneities in BUN (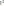
=81%), osmolality (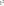
=78%), C-peptide (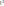
=77%), urine albumin (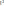
=77%), uric acid (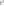
=66%), MMA (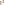
=58%), retinol (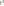
=57%), and heart disease (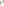
=53%). In general, despite obvious heterogeneities in some phenotypes, the effects of each phenotype were similar in different surveys.

## Discussion

In an analysis of evaluating 985 phenotypes in the NHANES using PheWAS, we found 18 phenotypes highly related to CKD, among which some are well-documented risk factors for CKD, but some are less researched previously, e.g., retinol, RDW, and C-peptide. The combination of identified phenotypes showed a well diagnostic efficacy for CKD. To our knowledge, the current study was the first to evaluate multiple phenotypes with CKD simultaneously and systematically to date, including environmental and clinical traits. Despite its exploratory nature, the PheWAS methodology provided a panoramic understanding of the multiple risk factors related to CKD, which may guide subsequent research, promote the diagnosis of CKD and imply potential mechanisms in the pathogenesis of CKD.

Our findings based on PheWAS offered a panoramic insight into the search for multiple factors of CKD at one time 17. All the identified phenotypes belong to clinical categories, and no environmental phenotype was validated, which indicated very little correlation between environmental phenotypes with CKD. We corroborated some acknowledged clinical phenotypes which have been proved to be associated with CKD in previous studies. BUN, a most discriminating and influential phenotype, has been considered as a marker of CKD and was used to calculate GFR 18. Hyperhomocysteinemia, hyperuricemia, hypoproteinemia, hyper-phosphatemia, methylmalonic acidemia, increased PTH, and serum osmolality level were significantly associated with CKD in prior studies 19-25. Besides, urinary albumin, as a marker of impairment of renal function, has been widely used in prognosis in CKD 26. Several studies revealed decreased levels of hemoglobin, hematocrit, and red cell count in patients with CKD due to deficiency of erythropoietin and shortening of red blood cell lifespan 27, 28. In addition, CKD is often accompanied by chronic diseases, such as hypertension and heart disease 29, 30. Our results provided further evidence for supporting these findings.

A notable finding was the significant association between serum retinol and CKD prevalence. Retinol showed well discrimination power in our RF model and had well diagnostic efficacy compared to other factors. However, previous work mainly focused on the association between retinol binding protein (RBP) with CKD, but less study was concerned with retinol levels 31, 32. Cabré *et al*. indicated that RBP, a transport protein of retinol, might be a marker of renal dysfunction in type 2 diabetic population 33. Indeed, Vannucchi *et al.* have verified the correlation between retinol level and RBP level 34. Hence, the relationship between retinol and CKD and the underlying mechanism deserve to be explored. There are some possible explanations to explain this observation. The impairment of excretion function in patients with CKD may result in the accumulation of RBP, thus causing an elevated retinol level 35; meanwhile, a positive feedback regulation to promote releasing of retinol-RBP-complex from the liver, may also lead to increased retinol level 36. This was supported by a cross-sectional study involving 105 children, which reported elevated levels of retinol in children with early CKD 37. We believe that retinol levels may reflect the renal function and could be a marker for CKD. Therefore, further validation of the association retinol with CKD is required to facilitate its application in the diagnosis and prediction of CKD.

Red cell distribution width, an index reflecting the volume variability of red blood cell, was another surprising finding with solid correlation with CKD. Recent evidence suggested that RDW was significantly associated with adverse clinical outcomes, such as mortality and cardiovascular disease 38, 39. However, poor study was found on the association between RDW and CKD. Our finding could have been generated by two possible mechanisms. RDW is highly correlated with oxidative stress and subsequent endothelial dysfunction, which are known as risk factors of CKD 40, 41. Meanwhile, metabolic disturbance of folic acid and vitamin B12, manifested as elevated RDW level, also contributes to the incidence of CKD 42.

We also verified the relationship between increased C-peptide concentration and incident CKD in our study. C-peptide, as a cleavage product of proinsulin, showed strong correlation with renal function in patients with diabetic nephropathy in a number of studies 43, 44. In an observational study involving 132 diabetics, Wong *et al*. reported a marked elevated concentration of serum C-peptide in those with end-stage renal failure 43. In the present study, we proved that phenomenon among population with CKD, and that not confined to diabetic nephropathy. Our observation was in good consistent with Adrian's study, which showed increased serum C-peptide level in both diabetic ESRD population and nondiabetic ESRD population 45. A possible explanation for this may be that kidney is responsible for the metabolism and excretion of C-peptide so a decline renal function may result in the accumulation of C-peptide.

Knowing the phenotypes highly related to CKD may improve the diagnostic efficacy for CKD. We hoped to develop a simplified diagnostic model capable of discriminating those with CKD. In our study, combination of identified phenotypes showed an excellent diagnostic value for CKD based on the ROC curve (AUROC=0.951 for full model and 0.914 for top 5 novel phenotypes). This diagnostic model will be helpful for clinicians to identify those who require intensive treatment and follow-up. Besides, most of these factors can be obtained from blood test and simple history inquiry, which will facilitate their application in clinical practice.

PheWAS, a data-driven approach capable of searching multiple phenotypes simultaneously, is able to generate hypothesis in patients with CKD for direct subsequent research. This methodology can overcome the limitation of selective reporting and eliminate factors with small effects 46. In order to obtain reliable results, we conducted internal cross-validation by random 50:50 split of the dataset into training set and testing set. We also validated the results by prioritizing the phenotypes based on RF model and Nagelkerke's Pseudo-R^2^, which indicated their differential performance and contribution for CKD, respectively. Besides, it should be noted that we used a large cross-sectional study with a large number of environmental and clinical phenotypes. The large sample size ensured the power and robustness of statistical analysis, and plenty of phenotypes enable a comprehensive correlation analysis.

Several limitations in our study should be considered. First, although some stronger correlations between phenotypes with CKD were found, they should be interpreted as correlative rather than causal. Further compelling evidence are needed to make causal inferences. Second, despite adjustment of covariates as much as possible, residual confounding might not be neglected and lead to bias inevitably in an observational study. Third, a total of 110 phenotypes fails to reach a minimum sample size of 500, therefore we are unable to include these phenotypes in our study which may result in missing important phenotypes. Finally, as NHANES is an observational study, the issue of reverse causality is unavoidable, which need further validation through Mendelian randomization or randomized trails. Besides, validation of the identified phenotypes in a prospective cohort may also provide persuasive evidence.

## Conclusion

Overall, we identified 18 phenotypes which demonstrate robust associations with CKD, including three novel phenotypes. The finding provided valid evidence for identifying of biomarkers for CKD and established an excellent diagnostic model with well diagnostic efficacy.

## Supplementary Material

Supplementary figures and tables.

## Figures and Tables

**Table 1:** Baseline characteristics of Participants with/without CKD in training set and testing set.

	Training dataset			Testing dataset		
	CKD	Non CKD	*P* value	CKD	Non CKD	*P* value
N	678	7229		677	7231	
Age, years	74.2±10.4	46.3±17.6	<0.001	73.8±11.2	46.4±17.5	<0.001
Female	351(51.8%)	3754(51.9%)	0.94	350(51.7%)	3789(52.4%)	0.73
Race						
White	470(69.3%)	3570(49.4%)	<0.001	469(69.3%)	3580(49.5%)	<0.001
Black	114(16.8%)	1401(19.4%)	0.11	116(17.1%)	1430(19.8%)	0.10
SES			<0.001			<0.001
Low	292(43.1%)	2805(38.8%)		297(43.9%)	2670(36.9%)	
Middle	244(36.0%)	2367(32.7%)		226(33.4%)	2363(32.7%)	
High	142(20.9%)	2057(28.5%)		154(22.7%)	2198(30.4%)	
BMI	28.2±5.8	28.5±6.5	0.24	28.7±5.9	28.5±6.3	0.41
Family smoker	91(13.4%)	1473(20.4%)	<0.001	92(13.6%)	1487(20.6%)	<0.001
Diabetes	190(28.0%)	701(9.7%)	<0.001	181(26.7%)	719(9.9%)	<0.001
Hypertension	463(68.3%)	2065(28.6%)	<0.001	466(68.8%)	2047(28.3%)	<0.001

CKD, chronic kidney disease; SES, Socioeconomic status; BMI, body mass index. Data are presented as mean ± SD or number (percentage).

**Table 2:** Adjusted Estimated Differences in CKD Associated with Phenotypes.

Phenotypes	Category	Training dataset				Testing dataset				RF ranking	Pseudo R^2^	AUROC
		N	CKD	OR (95%)	FDR	N	CKD	OR (95%)	FDR			
Hemoglobin	Blood routine	7898	678	0.64 (0.57, 0.71)	8.91e-14	7900	674	0.58 (0.53, 0.64)	8.12e-23	11	0.4283	0.619
Hematocrit	Blood routine	7898	678	0.64 (0.58, 0.71)	4.49e-14	7900	674	0.59 (0.53, 0.65)	1.62e-21	13	0.4282	0.609
Red cell count	Blood routine	7898	678	0.68 (0.62, 0.75)	3.67e-11	7900	674	0.62 (0.56, 0.68)	5.08e-19	8	0.4255	0.645
RDW	Blood routine	7898	678	1.29 (1.19, 1.40)	5.15e-07	7900	674	1.41 (1.30, 1.54)	1.51e-14	10	0.4201	0.674
BUN	Biochemistry	7907	678	5.05 (4.35, 5.89)	1.18e-94	7908	677	5.92 (5.04, 6.99)	3.61e-99	1	0.5593	0.877
Uric acid	Biochemistry	7907	678	2.57 (2.26, 2.93)	3.11e-43	7907	677	3.22 (2.83, 3.70)	1.50e-64	9	0.4774	0.729
Osmolality	Biochemistry	7907	678	1.93 (1.74, 2.14)	2.38e-32	7907	677	1.82 (1.65, 2.02)	2.39e-29	5	0.4466	0.751
PTH	Biochemistry	4137	427	2.09 (1.82, 2.40)	3.53e-22	4907	430	1.71 (1.50, 1.96)	1.34e-14	4	0.4880	0.716
Phosphorus	Biochemistry	7905	677	1.49 (1.35, 1.64)	4.66e-12	7908	677	1.38 (1.25, 1.53)	1.02e-09	14	0.4217	0.525
Serum Albumin	Biochemistry	7907	678	0.70 (0.63, 0.79)	2.10e-06	7908	677	0.72 (0.64, 0.80)	4.06e-09	6	0.4176	0.611
C-peptide	Biochemistry	2869	241	1.80 (1.50, 2.18)	8.54e-07	2857	219	2.40 (1.95, 2.97)	2.13e-15	16	0.4354	0.688
Homocysteine	Nutritional index	7891	677	3.15 (2.81, 3.55)	3.29e-79	7887	675	3.23 (2.88, 3.67)	8.05e-80	2	0.5064	0.877
Retinol	Nutritional index	7884	676	2.77 (2.45, 3.15)	2.02e-55	7879	673	3.03 (2.67, 3.45)	3.59e-64	3	0.4861	0.764
MMA	Nutritional index	5685	457	2.00 (1.79, 2.24)	1.09e-31	5779	456	2.25 (2.01, 2.53)	1.62e-43	7	0.4544	0.836
Urine Albumin	Urine test	7818	655	1.57 (1.44, 1.71)	6.03e-22	7824	645	1.64 (1.51, 1.79)	3.32e-28	12	0.4400	0.655
Chronic disease	Disease history	7907	678	1.69 (1.52, 1.88)	1.33e-19	7908	677	1.54 (1.39, 1.71)	6.10e-15	15	0.4283	0.760
Hypertension	Disease history	7907	678	2.28 (1.87, 2.78)	3.68e-13	7908	677	2.22 (1.82, 2.70)	1.51e-14	18	0.4252	0.701
Heart disease	Disease history	7907	678	2.20 (1.77, 2.72)	1.06e-09	7908	677	2.23 (1.80, 2.75)	6.26e-13	17	0.4214	0.631

RDW, red cell distribution width; BUN, blood urea nitrogen; PTH, parathyroid hormone; MMA, methylmalonic acid. Adjusted for sex, age, ethnicity, body mass index and socioeconomic status (SES) level.

## Abbreviations

PheWAS: phenotype-wide association study
NHANES: National Health and Nutrition Examination Survey
CKD: Chronic kidney disease
GFR: glomerular filtration rate
RDW: red cell distribution width
BUN: blood urea nitrogen
UA: uric acid
PTH: parathyroid hormone
HCY: homocysteine
MMA: methylmalonic acid
