# Supplementary material for: Identification of Novel Phenotypes Correlated with CKD: A Phenotype-Wide Association Study
Source: Int J Med Sci. 2022 Oct 31;19(13):1920–8. doi: 10.7150/ijms.63973 (PMC9682501; doi:10.7150/ijms.63973)
Supplement: Supplementary file 1 — Supplementary figures and tables. [file ijmsv19p1920s1.pdf]

**Text S1 Definition of chronic disease.**

According to the published description on NHANES database, chronic disease count was defined as the number of the following six conditions, including diabetes, coronary disease, hypertension, asthma, arthritis (rheumatoid arthritis, osteoarthritis), and 30 site-specific cancers.

Reference: Patel CJ, et al. A database of human exposomes and phenomes from the US National Health and Nutrition Examination Survey. Sci Data. 2016 Oct 25;3:160096.

**Table S1 Environmental and clinical phenotypes included in the PheWAS.**

| Category | Number | Number of Validations |
|---|---|---|
| Body measurements | 31 | 0 |
| Blood routine | 19 | 3 |
| Biochemistry | 76 | 7 |
| Nutritional status | 42 | 3 |
| Urine test | 78 | 1 |
| Infection status | 38 | 0 |
| Disease history | 39 | 3 |
| Drugs used | 204 | 0 |
| Lifestyle | 46 | 0 |
| Living condition | 14 | 0 |
| Dietary | 208 | 0 |
| Drug addiction | 14 | 0 |
| Dioxins | 29 | 0 |
| Polychlorinated biphenyls | 35 | 0 |
| Pesticides | 34 | 0 |
| Phenols | 9 | 0 |
| Polybrominated diphenyl ethers | 15 | 0 |
| Polyfluorinated compounds | 11 | 0 |
| Volatile organic compounds | 43 | 0 |

**Table S2 Logistic regressions show associations between retinol with CKD in subgroup analysis.**

| Subgroup | Events/Participants | Odds Ratio (95%CI) | P value | P for interaction |
|---|---|---|---|---|
| Gender | | | | 0.190 |
| Male | 7571/15815 | 3.06 (2.70-3.48) | <0.001 | |
| Female | 8244/15815 | 2.74 (2.42-3.10) | <0.001 | |
| Race | | | | 0.005 |
| White | 8089/15815 | 2.58 (2.31-2.90) | <0.001 | |
| Black | 3061/15815 | 3.79 (3.11-4.63) | <0.001 | |
| Others | 4665/15815 | 3.09 (2.52-3.80) | | |
| Age, years | | | | <0.001 |
| Age⩾46 | 8188/15815 | 2.60 (2.40-2.81) | <0.001 | |
| Age < 46 | 7627/15815 | 10.2 (6.23-16.56) | <0.001 | |
| BMI, $kg/m^2$ | | | | 0.022 |
| BMI⩾30 | 5217/15815 | 3.33 (2.83-3.91) | <0.001 | |
| BMI < 30 | 10598/15815 | 2.76 (2.48-3.07) | <0.001 | |
| Diabetes | | | | 0.291 |
| With diabetes | 1791/15815 | 3.06 (2.58-3.63) | <0.001 | |
| Free from diabetes | 14024/15815 | 2.76 (2.48-3.06) | <0.001 | |
| Hypertension | | | | 0.017 |
| With hypertension | 5041/15815 | 2.96 (2.64-3.31) | <0.001 | |
| Free from hypertension | 10774/15815 | 2.40 (2.06-2.79) | <0.001 | |

**Table S3 Logistic regressions show associations between RDW with CKD in subgroup analysis.**

| Subgroup | Events/Participants | Odds Ratio (95%CI) | P value | P for interaction |
|---|---|---|---|---|
| Gender | | | | 0.015 |
| Male | 7571/15815 | 0.91 (0.85-0.97) | <0.001 | |
| Female | 8244/15815 | 0.91 (0.85-0.97) | <0.001 | |
| Race | | | | 0.782 |
| White | 8089/15815 | 0.92 (0.86-0.98) | <0.001 | |
| Black | 3061/15815 | 0.88 (0.81-0.95) | <0.001 | |
| Others | 4665/15815 | | | |
| Age, years | | | | 0.679 |
| Age≥46 | 8188/15815 | 0.88 (0.82-0.93) | <0.001 | |
| Age < 46 | 7627/15815 | 0.97 (0.89-1.05) | <0.001 | |
| BMI, kg/$m^2$ | | | | 0.194 |
| BMI≥30 | 5217/15815 | 0.99 (0.91-1.08) | <0.001 | |
| BMI < 30 | 10598/15815 | 0.91 (0.82-1.00) | <0.001 | |
| Diabetes | | | | 0.396 |
| With diabetes | 1791/15815 | 0.89 (0.83-0.95) | <0.001 | |
| Free from diabetes | 14024/15815 | 0.94 (0.88-1.01) | <0.001 | |
| Hypertension | | | | 0.013 |
| With hypertension | 5041/15815 | 0.91 (0.85-0.96) | <0.001 | |
| Free from hypertension | 10774/15815 | 0.92 (0.84-1.00) | <0.001 | |

**Table S4 Logistic regressions show associations between C-peptide with CKD in subgroup analysis.**

| Subgroup | Events/Participants | Odds Ratio (95%CI) | P value | P for interaction |
|---|---|---|---|---|
| Gender | | | | 0.985 |
| Male | 7571/15815 | 0.91 (0.85-0.97) | <0.001 | |
| Female | 8244/15815 | 0.91 (0.85-0.97) | <0.001 | |
| Race | | | | 0.278 |
| White | 8089/15815 | 0.92 (0.86-0.98) | <0.001 | |
| Black | 3061/15815 | 0.88 (0.81-0.95) | <0.001 | |
| Others | 4665/15815 | | | |
| Age, years | | | | 0.023 |
| Age≥46 | 8188/15815 | 0.88 (0.82-0.93) | <0.001 | |
| Age < 46 | 7627/15815 | 0.97 (0.89-1.05) | <0.001 | |
| BMI, kg/m$^2$ | | | | 0.285 |
| BMI≥30 | 5217/15815 | 0.99 (0.91-1.08) | <0.001 | |
| BMI < 30 | 10598/15815 | 0.91 (0.82-1.00) | <0.001 | |
| Diabetes | | | | 0.298 |
| With diabetes | 1791/15815 | 0.89 (0.83-0.95) | <0.001 | |
| Free from diabetes | 14024/15815 | 0.94 (0.88-1.01) | <0.001 | |
| Hypertension | | | | 0.001 |
| With hypertension | 5041/15815 | 0.91 (0.85-0.96) | <0.001 | |
| Free from hypertension | 10774/15815 | 0.92 (0.84-1.00) | <0.001 | |

**Figure S1 Heatmap showing Pearson coefficient correlations between validated phenotypes**

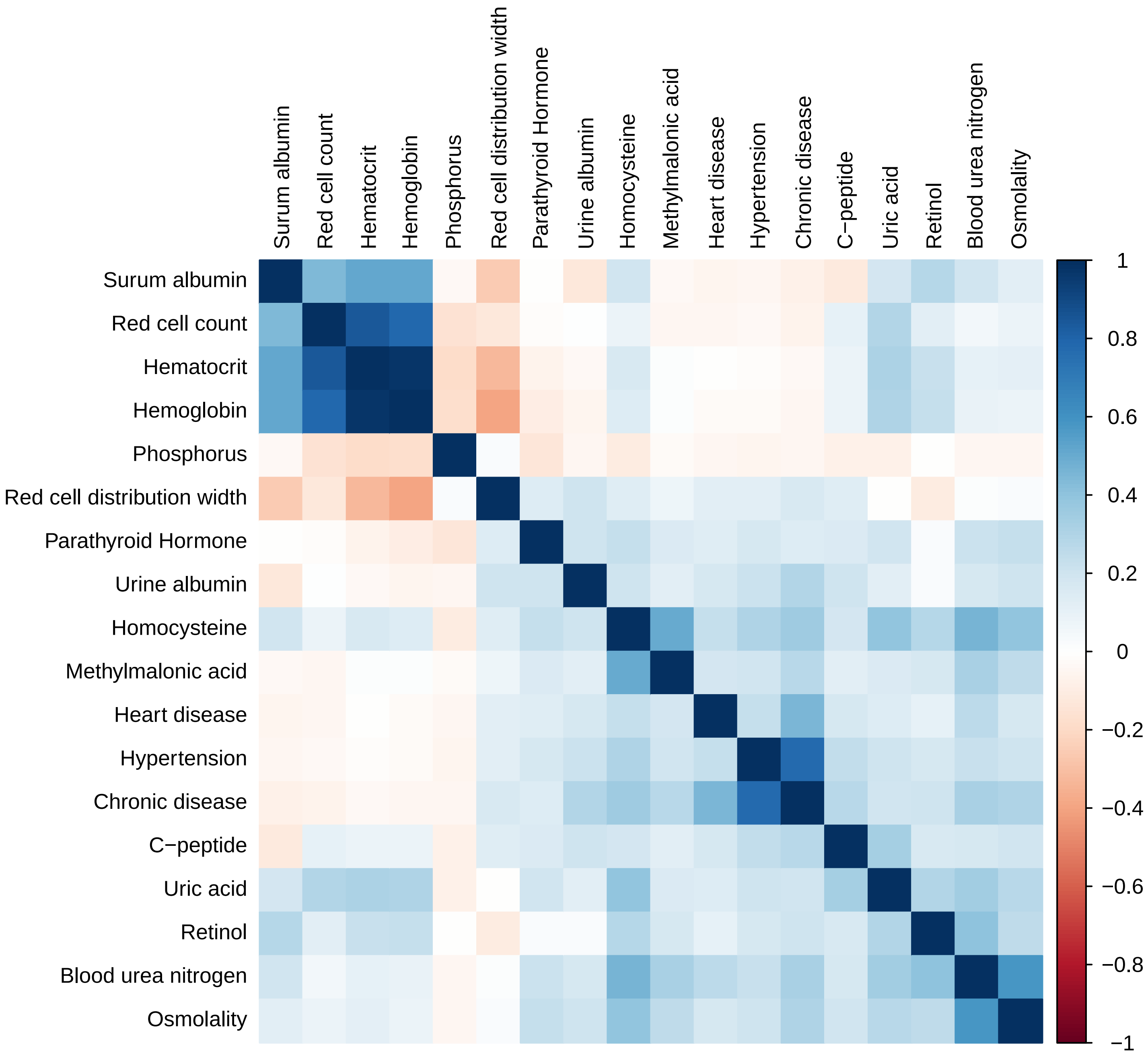

## Figure S2 ROC curves of all identified phenotypes

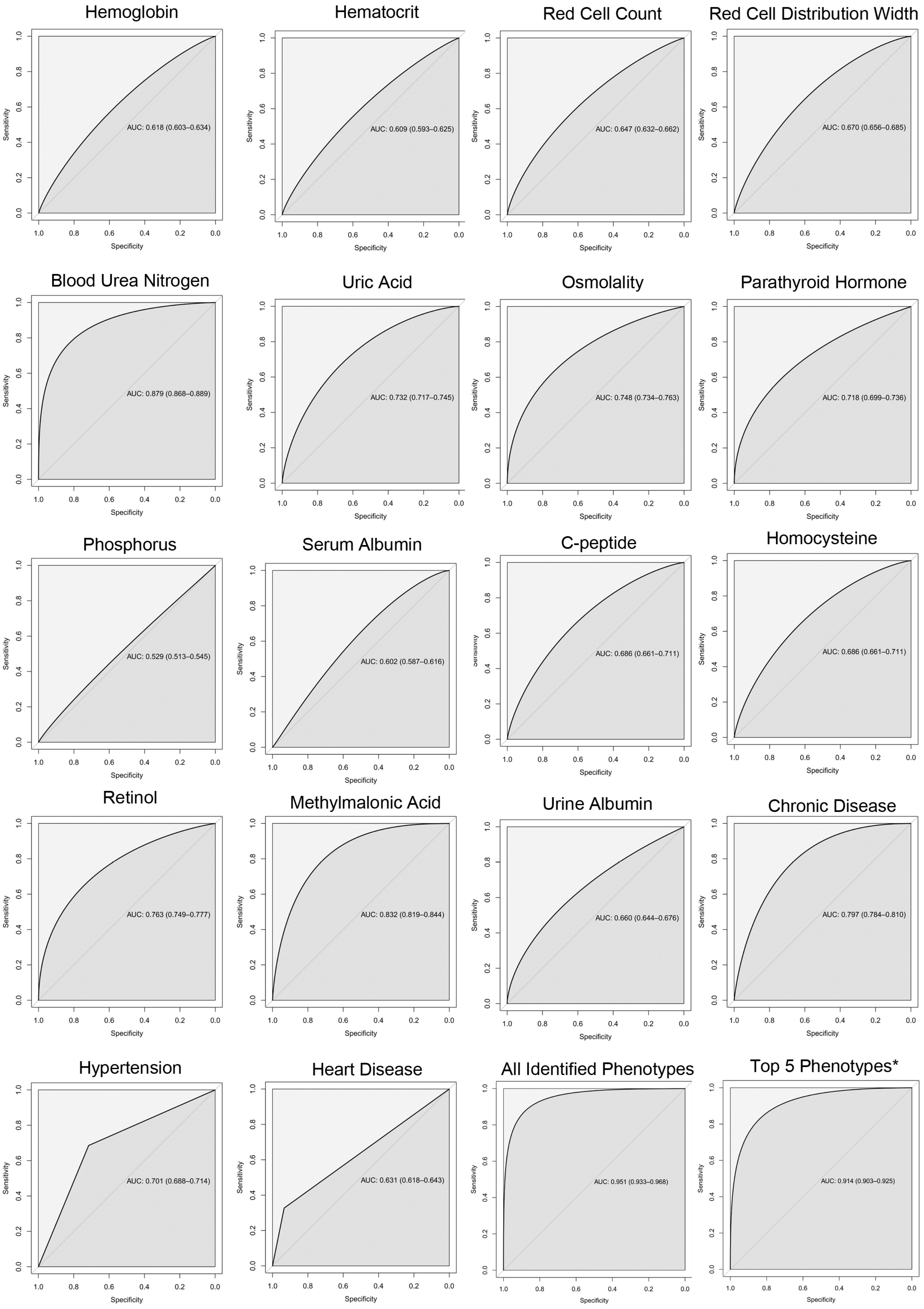


* Top 5 phenotypes: HCY, retinol, PTH, osmolality, serum albumin

Figure S3 Random-effect META-analyses of all identified phenotypes per survey

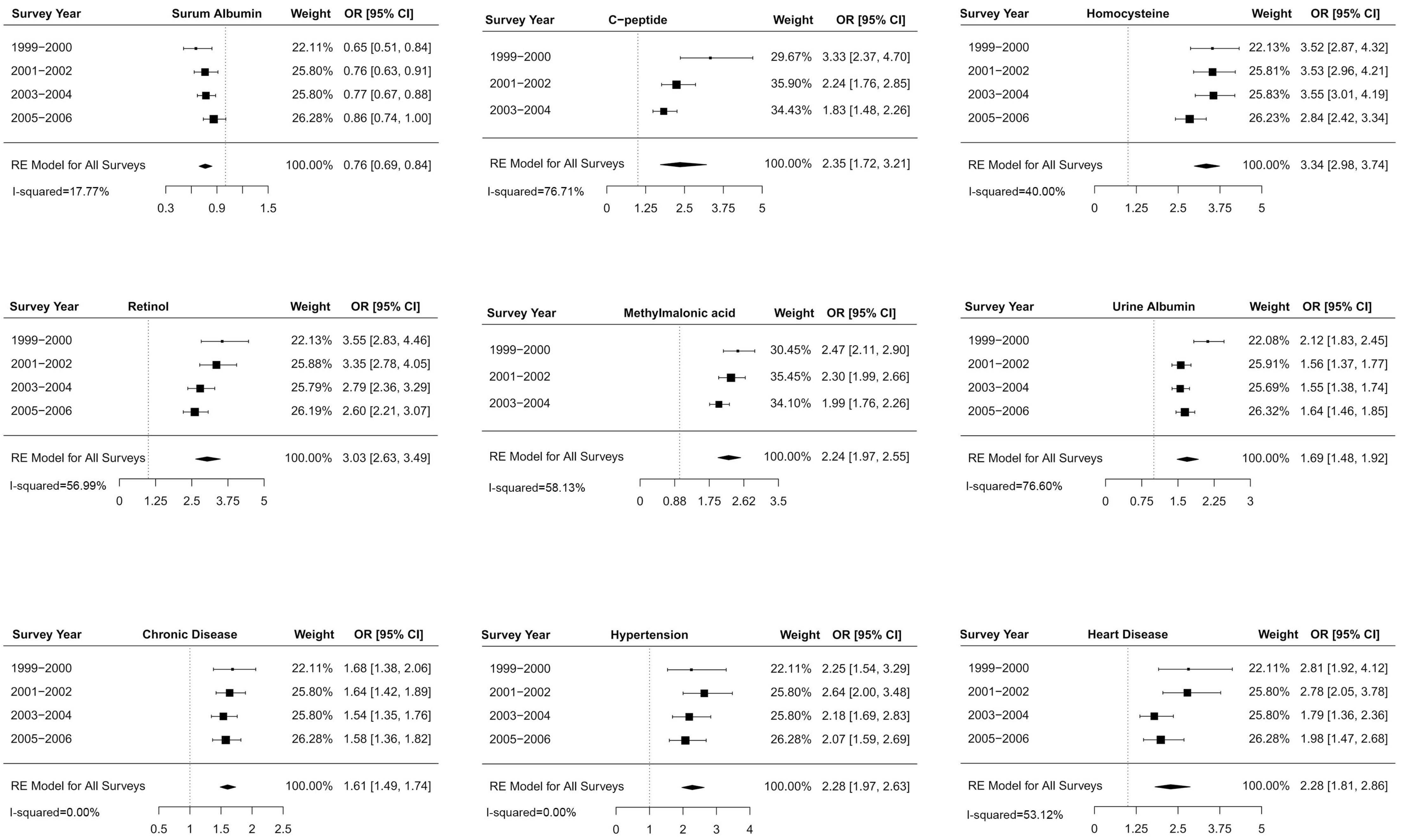

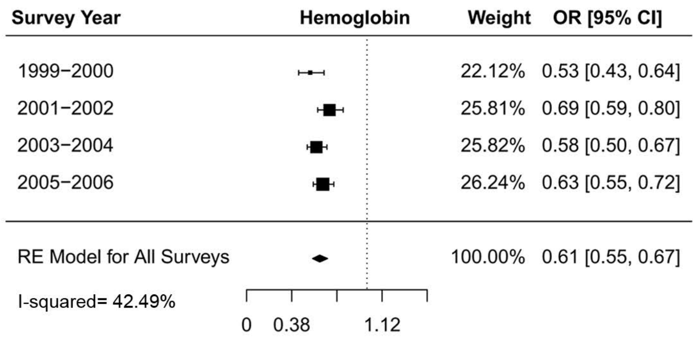
Survey Year
Hemoglobin
Weight
OR [95% CI]
1999−2000 22.12% 0.53 [0.43, 0.64]
2001−2002 25.81% 0.69 [0.59, 0.80]
2003−2004 25.82% 0.58 [0.50, 0.67]
2005−2006 26.24% 0.63 [0.55, 0.72]
RE Model for All Surveys 100.00% 0.61 [0.55, 0.67]
I-squared= 42.49%
0
0.38
1.12

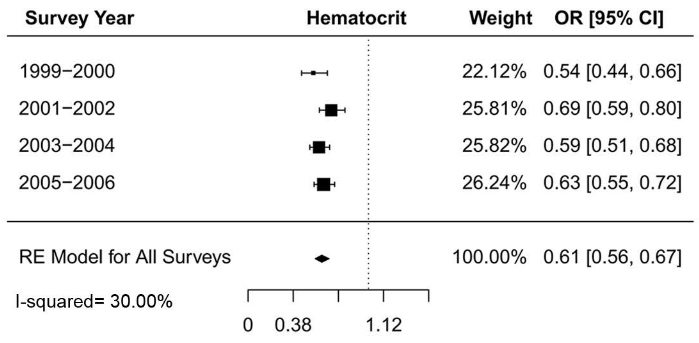
Survey Year
Hematocrit
Weight
OR [95% CI]
1999−2000 22.12% 0.54 [0.44, 0.66]
2001−2002 25.81% 0.69 [0.59, 0.80]
2003−2004 25.82% 0.59 [0.51, 0.68]
2005−2006 26.24% 0.63 [0.55, 0.72]
RE Model for All Surveys 100.00% 0.61 [0.56, 0.67]
I-squared= 30.00%
0
0.38
1.12

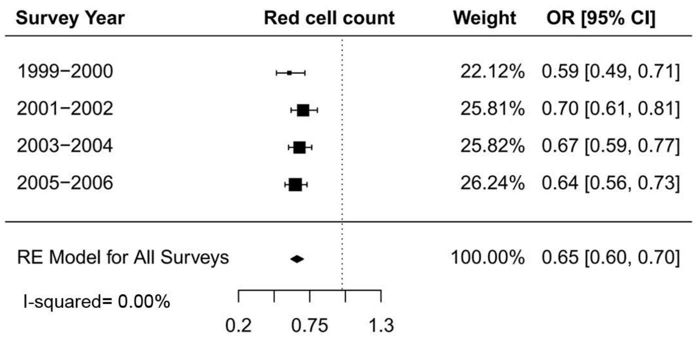
Survey Year
Red cell count
Weight
OR [95% CI]
1999−2000 22.12% 0.59 [0.49, 0.71]
2001−2002 25.81% 0.70 [0.61, 0.81]
2003−2004 25.82% 0.67 [0.59, 0.77]
2005−2006 26.24% 0.64 [0.56, 0.73]
RE Model for All Surveys 100.00% 0.65 [0.60, 0.70]
I-squared= 0.00%
0.2
0.75
1.3

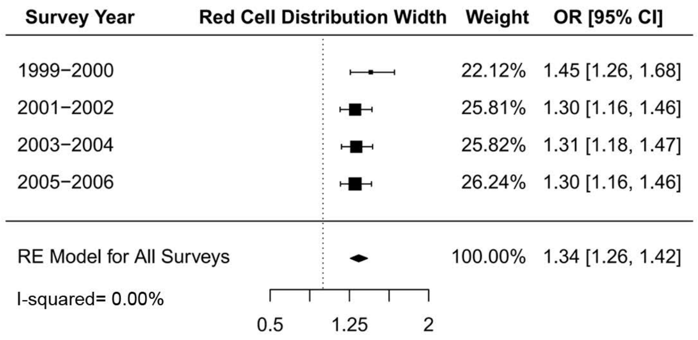
Survey Year
Red Cell Distribution Width
Weight
OR [95% CI]
1999−2000 22.12% 1.45 [1.26, 1.68]
2001−2002 25.81% 1.30 [1.16, 1.46]
2003−2004 25.82% 1.31 [1.18, 1.47]
2005−2006 26.24% 1.30 [1.16, 1.46]
RE Model for All Surveys 100.00% 1.34 [1.26, 1.42]
I-squared= 0.00%
0.5
1.25
2

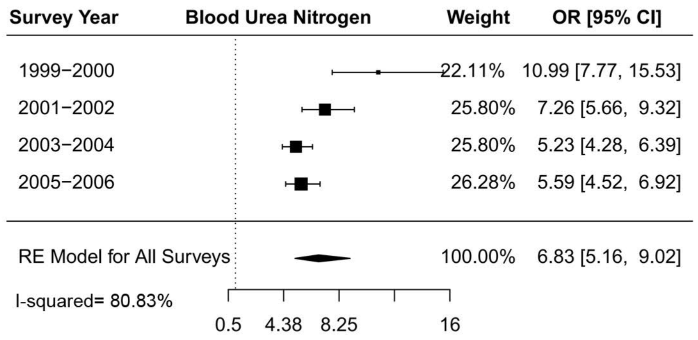
Survey Year
Blood Urea Nitrogen
Weight
OR [95% CI]
1999−2000 22.11% 10.99 [7.77, 15.53]
2001−2002 25.80% 7.26 [5.66, 9.32]
2003−2004 25.80% 5.23 [4.28, 6.39]
2005−2006 26.28% 5.59 [4.52, 6.92]
RE Model for All Surveys 100.00% 6.83 [5.16, 9.02]
I-squared= 80.83%
0.5
4.38
8.25
16

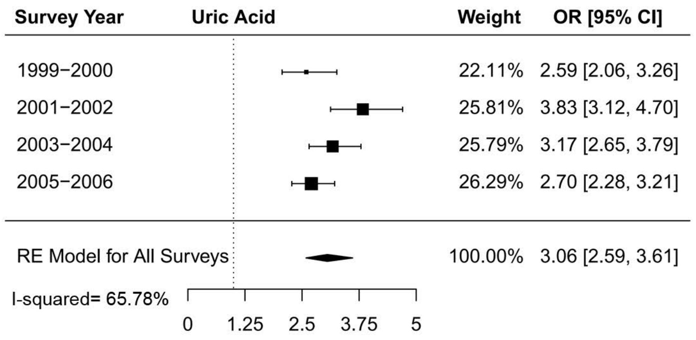
Survey Year
Uric Acid
Weight
OR [95% CI]
1999−2000 22.11% 2.59 [2.06, 3.26]
2001−2002 25.81% 3.83 [3.12, 4.70]
2003−2004 25.79% 3.17 [2.65, 3.79]
2005−2006 26.29% 2.70 [2.28, 3.21]
RE Model for All Surveys 100.00% 3.06 [2.59, 3.61]
I-squared= 65.78%
0
1.25
2.5
3.75
5

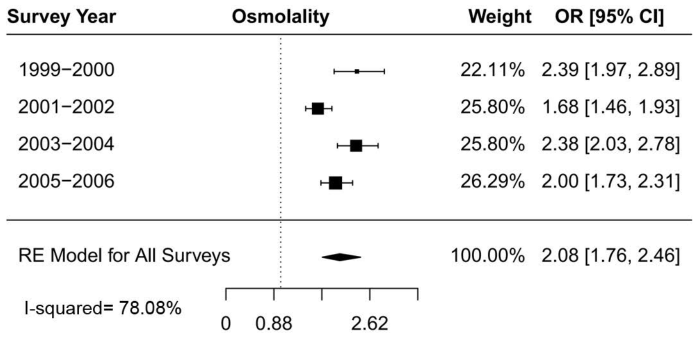
Survey Year
Osmolality
Weight
OR [95% CI]
1999−2000 22.11% 2.39 [1.97, 2.89]
2001−2002 25.80% 1.68 [1.46, 1.93]
2003−2004 25.80% 2.38 [2.03, 2.78]
2005−2006 26.29% 2.00 [1.73, 2.31]
RE Model for All Surveys 100.00% 2.08 [1.76, 2.46]
I-squared= 78.08%
0
0.88
2.62

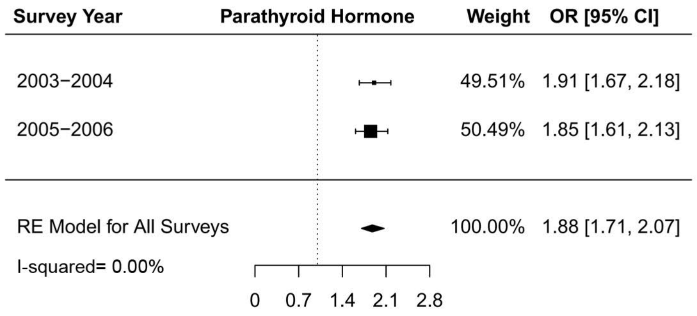
Survey Year
Parathyroid Hormone
Weight
OR [95% CI]
2003−2004 49.51% 1.91 [1.67, 2.18]
2005−2006 50.49% 1.85 [1.61, 2.13]
RE Model for All Surveys 100.00% 1.88 [1.71, 2.07]
I-squared= 0.00%
0
0.7
1.4
2.1
2.8

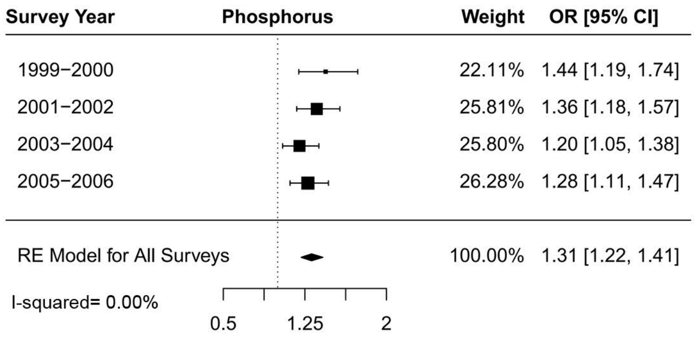
Survey Year
Phosphorus
Weight
OR [95% CI]
1999−2000 22.11% 1.44 [1.19, 1.74]
2001−2002 25.81% 1.36 [1.18, 1.57]
2003−2004 25.80% 1.20 [1.05, 1.38]
2005−2006 26.28% 1.28 [1.11, 1.47]
RE Model for All Surveys 100.00% 1.31 [1.22, 1.41]
I-squared= 0.00%
0.5
1.25
2
